# Diagnostic and prognostic value of baseline FDG PET/CT skeletal textural features in diffuse large B cell lymphoma

**DOI:** 10.1007/s00259-017-3899-6

**Published:** 2017-12-07

**Authors:** Nicolas Aide, Marjolaine Talbot, Christophe Fruchart, Gandhi Damaj, Charline Lasnon

**Affiliations:** 1 Nuclear Medicine Department, Caen University Hospital, Caen, France; 2 Normandie University, Caen, France; 3 INSERM 1086 ANTICIPE, Normandie University, Caen, France; 4 Haematology Institute, François Baclesse Cancer Centre, Caen, France; 5 Haematology Institute, Caen University Hospital, Caen, France; 6 Nuclear Medicine Department, François Baclesse Cancer Centre, Caen, France

**Keywords:** Diffuse large B cell lymphoma, FDG, PET, Bone marrow, Heterogeneity

## Abstract

**Purpose:**

Our purpose was to evaluate the diagnostic and prognostic value of skeletal textural features (TFs) on baseline FDG PET in diffuse large B cell lymphoma (DLBCL) patients.

**Methods:**

Eighty-two patients with DLBCL who underwent a bone marrow biopsy (BMB) and a PET scan between December 2008 and December 2015 were included. Two readers blinded to the BMB results visually assessed PET images for bone marrow involvement (BMI) in consensus, and a third observer drew a volume of interest (VOI) encompassing the axial skeleton and the pelvis, which was used to assess skeletal TFs. ROC analysis was used to determine the best TF able to diagnose BMI among four first-order, six second-order and 11 third-order metrics, which was then compared for diagnosis and prognosis in disease-free patients (BMB−/PET-) versus patients considered to have BMI (BMB+/PET-, BMB−/PET+, and BMB+/PET+).

**Results:**

Twenty-two out of 82 patients (26.8%) had BMI: 13 BMB−/PET+, eight BMB+/PET+ and one BMB+/PET-. Among the nine BMB+ patients, one had discordant BMI identified by both visual and TF PET assessment. ROC analysis showed that SkewnessH, a first-order metric, was the best parameter for identifying BMI with sensitivity and specificity of 81.8% and 81.7%, respectively. SkewnessH demonstrated better discriminative power over BMB and PET visual analysis for patient stratification: hazard ratios (HR), 3.78 (*P* = 0.02) versus 2.81 (*P* = 0.06) for overall survival (OS) and HR, 3.17 (*P* = 0.03) versus 1.26 (*P* = 0.70) for progression-free survival (PFS). In multivariate analysis accounting for IPI score, bulky status, haemoglobin and SkewnessH, the only independent predictor of OS was the IPI score, while the only independent predictor of PFS was SkewnessH.

**Conclusion:**

The better discriminative power of skeletal heterogeneity for risk stratification compared to BMB and PET visual analysis in the overall population, and more specifically in BMB−/PET- patients, suggests that it can be useful to identify diagnostically overlooked BMI.

## Introduction

Diffuse large B-cell lymphoma (DLBCL) accounts for 30% to 58% of the non-Hodgkin’s lymphoma series [1]. Positron emission tomography coupled with computed tomography (PET/CT) has become the standard non-invasive examination for the initial staging of DLBCL [2, 3]. It improves the accuracy of staging compared to CT and leads to stage migration in 10 to 30% of patients. Consequently, fewer patients are undertreated or overtreated [4]. In regard to bone marrow involvement (BMI), focal bone marrow FDG uptake with or without increased diffuse uptake is more sensitive than bone marrow biopsy (BMB) but can overlook low-volume diffuse involvement of 10% to 20% of the marrow and discordant lymphoma (small cells) [5–7]. The proportion of patients in whom BMB is positive while FDG PET/CT is negative for BMI has been estimated at 3.1% [8]. This could be explained by the lack of consensus on whether diffuse bone marrow FDG uptake should be regarded as a positive or a negative finding, as highlighted in a recent meta-analysis on PET/CT for the detection of BMI in DLBCL [8]. Moreover, Paone et al. [9] found that PET-CT was more sensitive for the detection of concordant BM involvement (large cells) than discordant BM involvement (small cells). Therefore, in most cases, positive PET/CT is usually sufficient to designate advanced-stage disease, and BMB is not required. However, if the scan is negative, a BMB could be indicated to identify involvement relevant for a clinical trial or patient management and especially discordant histology.

Regarding the prognostic impact of BM status as determined by FDG PET/CT, discrepant findings have been published. In a retrospective study including 133 patients, Berthet et al. demonstrated that PET was an independent predictor for progression-free survival (PFS) but not overall survival (OS) in a multivariate analysis [5], whereas Hong et al. found no differences in PFS or OS between PET-positive and PET-negative patients [10]. A third study stated that the outcome of patients with positive PET findings was comparable to that of other patients with stage IV disease without positive BMB [6]. Finally, the latest study to date showed that bone marrow status assessed by baseline PET is an independent predictor of OS with worse survival outcomes in patients with BMI in patients staged IV. These conflicting results suggest that further research is needed.

There is currently growing interest in oncology in using alternatives to visual or semi-quantitative PET assessment that are based on SUV metrics as diagnostic and prognostic indicators or probabilistic indicators of response to treatment. Until now, these metrics (textural features, TFs) were applied only to primary tumours and studied mainly as predictors of treatment response or prognostic factors [11, 12]. We assume that in the framework of newly diagnosed DLBCL, FDG PET TFs may provide a more comprehensive quantitative assessment of bone involvement, in particular in doubtful patients displaying diffuse and heterogeneous skeleton uptake. Thus, the aim of this study was to evaluate (1) the value of textural features (TFs) for the diagnosis of bone involvement, especially in diffuse and discordant BMI, and (2) the prognostic value of TF-based bone marrow assessment in patients with newly diagnosed DLBCL.

## Materials and methods

### Population

All patients diagnosed with diffuse large B cell lymphoma and who had a bone marrow biopsy (BMB) were retrospectively included from December 2008 to December 2015. In accordance with European regulations, French observational studies without any additional therapy or monitoring procedure do not need the approval of an ethical committee. Nonetheless, we sought approval to collect data for our study from the national committee for data privacy, the National Commission on Informatics and Liberty (CNIL), with registration n°2,081,250 v 0.

### PET acquisition and reconstruction parameters

After a 15-min rest in a warm room, patients who had been fasting for 6 h were injected intravenously with 18F-FDG. Height, weight, the injected dose, the capillary glycaemia at the injection time and the exact delay between injection and the start of the acquisition were recorded for each patient. Body mass index was used to separate overweight and obese patients (≥ 25 kg/m^2^) from low to normal-weight patients (< 25 kg/m^2^) for whom a longer time per bed position was used.

All PET imaging studies were performed on a Biograph TrueV (Siemens Medical Solutions) with a 6-slice spiral CT (Computed Tomography) component. Additional technical details regarding the system and PSF reconstruction can be found elsewhere [13, 14]. CT acquisition was performed first with the following parameters: 60 mAs, 130 kVp, pitch 1 and 6 × 2 mm collimation. Subsequently, the PET emission acquisition was performed in 3D-mode. For low to normal-weight patients and overweight to obese patients, the durations were 2 min 40 s and 3 min 40 s, respectively. Patients were scanned from the skull to the mid-thighs. All examinations were reconstructed using an OSEM algorithm with point spread function (PSF) modelling (HD; TrueX, Siemens Medical Solution) with three iterations and 21 subsets without filtering. The matrix size was 168 × 168 voxels, resulting in isotropic voxels of 4.07 × 4.07 × 4.07 mm^3^. Scatter and attenuation corrections were applied.

### PET visual interpretation

PET examinations were reviewed using MIM (MIM software, Cleveland, OH, USA, version 5.6.5). Two experienced readers, blinded to BMB results, visually assessed the bone status of each patient. PET/CT examinations were considered to be positive in cases of one or several bone focal uptakes on PET images with or without bone lesion on CT images. Diffuse and/or heterogeneous skeleton uptake was not considered a positive finding. In case of discrepancy, the examination was conjointly reviewed to reach a consensus.

### Extraction of PET bone textural features

**Fig. 1:**
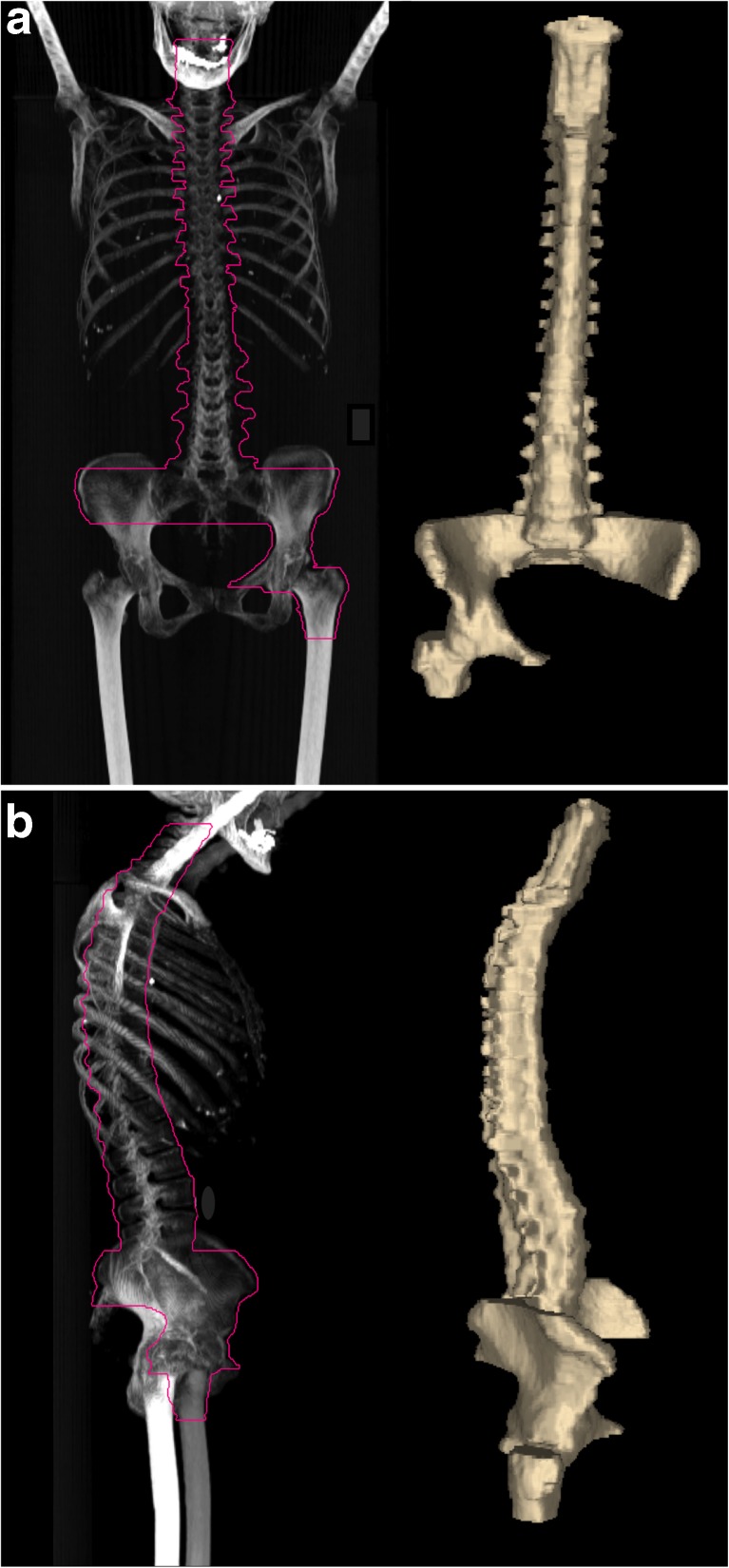
Representative example of a final skeletal volume of interest. Frontal (a) and lateral (b) maximum intensity projection (MIP) views

The following procedure was made in duplicate by one junior and one senior PET reader. For each examination, a preliminary volume of interest (VOI) involving the axial skeleton was drawn using CT densities [Hounsfield units (HU) >150]. To obtain a single volume of interest (VOI) encompassing the spine and half of the pelvis, the VOI was manually adapted to exclude all unwanted bone areas. The chosen pelvic area was the one reported to be the site of BMB in the medical report, when mentioned. Otherwise, the right part of the pelvis was arbitrarily chosen. The only exception was the presence of a hip prosthesis, for which the contralateral pelvic area was chosen to avoid PET attenuation correction artefacts. The final CT VOI was then transferred to PET images and checked for all possible lymph node areas of increased FDG uptake in the vicinity of the skeleton (especially in the retroperitoneum) that could affect texture features because of a spill-over effect [15]. Areas of contiguous bone involvement were also manually excluded. Finally, the VOI was saved in DICOM-RT structure format (Fig. 1) so that it could be processed on LIFEx (version 2.0), third party freeware developed by Buvat and co-workers [16, 17] (www.lifexsoft.org).

Each PET dataset and corresponding VOI were loaded into LifeX software to extract the following:

- First order metrics extracted from histogram: SkewnessH, KurtosisH, EntropyH and EnergyH.
- Second order metrics calculated from co-occurrence matrices: homogeneity, energy, contrast, correlation, entropy and dissimilarity. Index values were the average of indexes over the 13 directions in space.
- Third order metrics calculated from size-zone matrices: SZE (Short-Zone Emphasis), LZE (Long-Zone Emphasis), LGZE (Low Grey-level Zone Emphasis), HGZE (High Grey-LevelZone Emphasis), SZLGE (Short-Zone Low Grey-level Emphasis), SZHGE (Short-Zone High Grey-level Emphasis), LZLGE (Long-Zone Low Grey-level Emphasis), LZHGE (Long-Zone High Grey-level Emphasis), GLNU (Grey-Level Non-Uniformity for zone) ZLNU (Zone Length Non-Uniformity) and ZP (Zone Percentage). Index values were calculated using a single co-occurrence matrix taking into account all 13 spatial directions simultaneously [18, 19].

### Statistical analysis

Quantitative data are presented as the mean ± standard deviation (SD) or median (interquartile range) when appropriate. Characteristics of populations were compared by using Fischer’s exact tests or Chi-square tests for discrete variables and Mann-Whitney tests for continuous variables.

The agreement of VOIs and SkewnessH values between observers was evaluated by means of linear regressions, volume concordance indexes (Dice and Jaccard indexes) and Cohen’s kappa. Kappa (κ) value was reported using the benchmarks of Landis and Koch [20]. Volume concordance indexes were computed as follows:

$$ {\displaystyle \begin{array}{l} Diceindex=\frac{2\times \left({ VO I}_1\cap { VO I}_2\right)}{VO{I}_1+{ VO I}_2}\\ {} Jaccardindex=\frac{VO{I}_1\cap { VO I}_2}{VO{I}_1\cup { VO I}_2}\end{array}} $$

where ∩ and ∪ represent the intersection and the union of two VOIs, respectively.

For receivers operating characteristic (ROC) and survival analyses, BMB and PET results were taken as the gold standard. BMB−/PET- patients were considered disease-free patients (disease- patients) whereas BMB+/PET-, BMB−/PET+ and BMB+/PET+ patients were considered to have BMI (disease + patients). Receivers operating characteristic (ROC) analyses [21] were used to define area under the curve (AUC), Youden indexes and optimal cut-off values of each metric for the diagnosis of BMI.

Survival analysis was performed using univariate and multivariate Cox regression models. OS and PFS univariate survival functions were calculated by using Kaplan-Meyer survival analyses with log-rank tests to compare survival curves. For specific overall survival (OS), the end-point was defined as the time from diagnosis to the date of death from the lymphoma disease (lymphoma itself or treatment side-effects). For progression-free survival (PFS) the end-point was defined as the time from diagnosis to the point of relapse or progression.

Further ROC and survival analyses were conducted using overall survival (OS) and progression-free survival (PFS) data as the reference standard.

Statistical significance was considered at *p* < 0.05. Data were analysed using Graphpad Prism and MedCalc (MedCalc Software, Ostend, Belgium) software.

## Results

### Population characteristics

The baseline examinations of 84 patients were included. Two patients were excluded because of non-contributive BMB results. The population’s characteristics (*n* = 82) are summarised in Table 1. One PET examination (1.2%) did not fulfil the EANM procedure guidelines for tumour imaging [22]. The mean capillary glycaemia was 1.00 ± 0.20 g/l. The mean injected dose was 4.00 ± 0.29 MBq/kg, and the mean post injection imaging time was 62 ± 5 min. None of the patients had a history of inflammatory osteoarthritis. As the first line of treatment, patients had either a R-CHOP (69 patients) or a R-ACVBP (13 patients) regimen.

**Table 1:** Patients’ characteristics

Characteristics	Total population (*n* = 82)	SkewnessH ≤1.26 (*n* = 53)	SkewnessH >1.26 (*n* = 29)	P value
Age (years), *median (range)*	64 (20 – 88)	63 (20 – 88)	65.0 (43 – 80)	0.2002
Sex, *n (%)*				
Male	44 (53.7)	29 (54.7)	15 (51.7)	0.8206
Female	38 (46.3)	24 (45.3)	14 (48.3)	
B symptoms, *n (%)*				
Yes	17 (20.7)	10 (18.9)	7 (24.1)	0.5810
No	65 (79.3)	43 (81.1)	22 (75.9)	
Ann Arbor stage, *n (%)*				
I	8 (9.8)	8 (15.1)	0 (0.0)	<0.0001
II	18 (21.9)	17 (32.1)	1 (3.4)	
III	19 (23.2)	15 (28.3)	4 (13.8)	
IV	37 (45.1)	13 (24.5)	24 (82.8)	
Bulky mass, *n (%)*				
Yes	34 (41.5)	16 (30.2)	18 (62.1)	0.0093
No	48 (58.3)	37 (69.8)	11 (37.9)	
IPI score, n (%)				
0	20 (24.4)	20 (37.7)	0 (0.0)	<0.0001
1	21 (25.6)	16 (30.2)	5 (17.2)	
2	18 (22.0)	10 (18.9)	8 (27.6)	
3	21 (25.6)	6 (11.3)	15 (51.7)	
4	1 (1.2)	1 (1.9)	0 (0.0)	
5	1 (1.2)	0 (0.0)	1 (3.5)	
Hb (g/dL), *mean (SD)*	12.6 (2.08)	13.11 (2.15)	11.54 (1.57)	0.0003
WBC (G/L), *mean (SD)*	7.45 (2.84)	7.57 (2.94)	7.21 (2.64)	0.9150
Lymphocytes (G/L), *mean(SD)*	1.41 (1.09)	1.60 (1.25)	1.05 (1.60)	0.0158
LDH (UI/l), *mean (SD)*	356.17 (254.78)	308.5 (198.6)	442.7 (323.0)	0.0199

*IPI* international prognostic score, *Hb* haemoglobin, *WBC* white blood cells, *LDH* lactate dehydrogenase

### Diagnostic value of textural features for bone involvement at baseline staging

Among 82 patients, 22 (26.8%) were diagnosed with bone narrow involvement: 13 BMB−/PET+, eight BMB+/PET+ and one BMB+/PET-. Among the nine BMB+ patients, one (11.1%) had discordant bone involvement identified by both visual and TF PET assessments.

Textural feature ROC analyses for the diagnosis of bone involvement were highly statistically significant for all the first-order parameters with *p* values <0.0001. Among second-order and third-order parameters, two parameters over six (contrast and correlation) and five parameters over 11 (SZE, HGZE, SZHGE, LZHGE, ZLNU) were found to have statistically significant ROC analyses, respectively (Table 2). The parameter displaying the highest Youden index (J = 0.6348) and area under the curve (AUC = 0.820) was SkewnessH. ROC analyses with OS and PFS as reference standard are displayed in the Tables 3 and 4 as well as corresponding univariate survival analyses.

**Table 2:** ROC analyses for the diagnosis of bone involvement and corresponding univariate OS and PFS survival analyses

	ROC analyses							Univariate OS analyses			Univariate PFS analyses		
Parameters	AUC	95% CI	Youden index	Cut-off value	Sensitivity	Specificity	P value	HR	95%CI	P value	HR	95% CI	P value
BMB/PET reference													
Disease + *	–	–	–	–	–	–	–	2.81	0.78-10.15	0.062	1.26	0.36-4.34	0.700
First Order metrics													
SkewnessH	0.821	0.721–0.897	0.6348	>1.26	81.8	81.7	<0.0001	3.78	1.15-12.44	0.019	3.17	1.00-10.04	0.032
KurtosisH	0.813	0.712–0.891	0.6091	>4.89	90.9	70.0	<0.0001	3.47	1.12-10.79	0.047	2.77	0.93-8.26	0.077
EntropyH	0.787	0.682–0.870	0.5545	≤1.32	95.4	60.0	<0.0001	4.22	1.36-13.14	0.043	1.99	0.67-5.90	0.244
EnergyH	0.800	0.697–0.880	0.5227	>0.07	77.3	75.0	<0.0001	3.18	0.99-10.16	0.046	2.71	0.88-8.34	0.068
Second Order metrics													
Homogeneity	0.542	0.429–0.653	0.1364	≤0.53	13.6	100.0	0.5739	–	–	–	–	–	–
Energy	0.545	0.432–0.656	0.1197	≤0.02	13.6	98.3	0.5441	–	–	–	–	–	–
Contrast	0.669	0.557–0.769	0.3424	>2.88	40.9	93.3	0.0230	3.08	0.60-15.74	0.053	1.17	0.24-5.75	0.838
Correlation	0.745	0.637–0.835	0.4394	>0.75	77.3	66.7	0.0002	1.98	0.63-6.23	0.235	1.16	0.39-3.49	0.787
Entropy	0.609	0.495–0.715	0.2227	>1.45	77.3	45.0	0.1262	–	–	–	–	–	–
Dissimilarity	0.606	0.492–0.713	0.2273	>1.39	22.7	100.0	0.1583	–	–	–	–	–	–
Third Order metrics													
SZE	0.760	0.653–0.858	0.4667	>0.50	50.0	96.7	0.0001	4.22	0.84-21.11	0.007	1.84	0.38-8.80	0.346
LZE	0.631	0.517–0.735	0.2394	≤95,159.28	27.3	96.7	0.0681	–	–	–	–	–	–
LGZE	0.581	0.467–0.690	0.1742	≤0.09	59.1	58.3	0.2695	–	–	–	–	–	–
HGZE	0.768	0.662–0.854	0.5121	>89.84	54.5	96.7	<0.0001	4.05	0.83-19.75	0.009	1.72	0.37-7.95	0.402
SZLGE	0.584	0.470–0.692	0.1379	≤0.03	45.4	68.3	0.2507	–	–	–	–	–	–
SZHGE	0.778	0.673–0.862	0.4955	>46.23	54.5	95.0	<0.0001	3.42	0.77-15.31	0.025	2.21	0.51-9.51	0.174
LZLGE	0.574	0.460–0.683	0.1848	≤38,867.89	81.8	36.7	0.3131	–	–	–	–	–	–
LZHGE	0.687	0.575–0.785	0.3409	≤4,661,869.17	59.1	75.0	0.0068	1.40	0.42-4.62	0.566	1.76	0.55-5.60	0.302
GLNU	0.542	0.428–0.652	0.1833	≤153.40	50.0	68.3	0.5944	–	–	–	–	–	–
ZLNU	0.720	0.610–0.813	0.3924	>694.77	40.9	98.3	0.0012	2.72	0.67-11.00	0.074	1.13	0.30-4.30	0.850
ZP	0.628	0.514–0.732	0.3015	>0.04	31.8	98.3	0.0782	–	–	–	–	–	–

*BMB+/PET+, BMB+/PET- and BMB−/PET+ patients

**Table 3:** ROC analyses for OS and corresponding univariate OS survival analyses

	ROC analyses							Univariate OS analyses		
Parameters	AUC	95% CI	Youden index	Cut-off value	Sensitivity	Specificity	P value	HR	95% CI	P value
First Order metrics										
SkewnessH	0.749	0.641–0.839	0.3952	>1.50	66.7	72.9	0.0005	4.29	1.27-14.5	0.0094
KurtosisH	0.753	0.645–0.842	0.4595	>4.72	91.7	54.3	<0.0001	10.35	3.34-32.12	0.0053
EntropyH	0.749	0.641–0.838	0.4524	≤1.11	66.7	78.6	0.0001	5.60	1.55-20.22	0.0015
EnergyH	0.749	0.641–0.838	0.4381	>0.09	66.7	77.1	<0.0001	5.21	1.47-18.45	0.0026
Second Order metrics										
Homogeneity	0.514	0.401–0.626	0.1143	≤0.65	50.0	38.6	0.8847	–	–	–
Energy	0.524	0.411–0.635	0.0714	≤0.04	50.0	57.14	0.8022	–	–	–
Contrast	0.638	0.524–0.741	0.3429	>2.63	50.0	84.3	0.1734	–	–	–
Correlation	0.564	0.450–0.673	0.1810	>0.74	66.7	51.4	0.5126	–	–	–
Entropy	0.557	0.443–0.666	0.1833	>1.53	58.3	60.0	0.5508	–	–	–
Dissimilarity	0.587	0.473–0.695	0.2286	>1.04	50.0	72.9	0.3660	–	–	–
Third Order metrics										
SZE	0528	0.415–0.639	0.3024	>0.50	41.7	88.6	0.8026	–	–	–
LZE	0.540	0.427–0.651	0.1643	≤113,425.08	25.0	91.4	0.6808	–	–	–
LGZE	0.595	0.480–0.702	0.1881	≤0.14	91.7	27.1	0.2746	–	–	–
HGZE	0.683	0.571–0.782	0.3452	>112.02	41.7	92.9	0.0264	–	–	–
SZLGE	0.597	0.483–0.704	0.1786	≤0.04	75.0	42.9	0.2476	–	–	–
SZHGE	0.692	0.580–0.789	0.3452	>75.64	41.7	92.9	0.0149	6.24	0.97-40.22	0.0003
LZLGE	0.555	0.441–0.665	0.1881	≤51,423.79	91.7	27.1	0.5290	–	–	–
LZHGE	0.520	0.407–0.632	0.1916	≤3,741,969.49	33.3	82.9	0.8469	–	–	–
GLNU	0.517	0.404–0.629	0.1690	≤160.4	58.3	58.6	0.8683	–	–	–
ZLNU	0.613	0.499–0.719	0.2738	>564.67	41.7	85.7	0.2705	–	–	–
ZP	0.570	0.456–0.479	0.1786	>0.04	25.0	92.9	0.4554	–	–	–

**Table 4:** ROC analyses for PFS and corresponding univariate PFS survival analyses

	ROC analyses							Univariate PFS analyses		
Parameters	AUC	95% CI	Youden index	Cut-off value	Sensitivity	Specificity	P value	HR	95% CI	P value
First Order metrics										
SkewnessH	0.701	0.589–0.797	0.3735	>1.16	69.2	68.1	0.0081	3.91	1.26-12.10	0.0142
KurtosisH	0.676	0.563–0.775	0.3835	>9.9	61.5	76.8	0.0353	4.35	1.27-14.81	0.0048
EntropyH	0.651	0.537–0.753	03110	≤1.15	61.5	69.6	0.0836	–	–	–
EnergyH	0.633	0.520–0.737	0.3255	>0.08	61.5	71.0	0.1380	–	–	–
Second Order metrics										
Homogeneity	0.552	0.439–0.662	0.1616	>0.65	53.8	62.3	0.5766	–	–	–
Energy	0.570	0.456–0.679	0.1572	>0.05	46.1	69.6	0.4523	–	–	–
Contrast	0.502	0.389–0.614	0.1761	≤1.54	53.8	63.8	0.9863	–	–	–
Correlation	0.502	0.389–0.614	0.1226	≤0.74	38.5	49.3	0.9863	–	–	–
Entropy	0.571	0.457–0.680	0.2441	≤1.4	46.1	78.3	0.4670	–	–	–
Dissimilarity	0.552	0.439–0.662	0.1706	≤0.94	69.2	47.8	0.5721	–	–	–
Third Order metrics										
SZE	0.556	0.442–0.666	0.1282	>0.48	46.1	66.7	0.5403	–	–	–
LZE	0.535	0.422–0.646	0.2879	>533,174.76	46.1	82.6	0.7278	–	–	–
LGZE	0.515	0.402–0.627	0.1728	≤0.18	76.9	5.8	0.8814	–	–	–
HGZE	0.546	0.432–0.567	0.1817	>77.99	38.5	79.7	0.6244	–	–	–
SZLGE	0.504	0.392–0.617	0.1293	>0.07	23.1	89.9	0.9636	–	–	–
SZHGE	0.574	0.460–0.683	0.4166	>42.90	38.5	82.6	0.4166	–	–	–
LZLGE	0.536	0.423–0.647	0.7175	>42,917.4	46.1	73.9	0.7175	–	–	–
LZHGE	0.565	0.451–0.674	0.2040	≤5,919,722.88	76.9	43.5	0.4494	–	–	–
GLNU	0.502	0.389–0.614	0.1839	≤141.57	7.7	73.9	0.9849	–	–	–
ZLNU	0.511	0.398–0.623	0.1951	≤376.93	61.5	58.0	0.9059	–	–	–
ZP	0.517	0.404–0.629	0.0624	>0.10	7.7	98.6	0.8399	–	–	–

Linear regressions showed significant association between SkewnessH values and haemoglobin and LDH values with *r*^2^ values equal to 0.10 (*p* = 0.005) and 0.08 (*p* = 0.01), respectively. There was no significant association between SkewnessH values and lymphocytes level (*r*^2^ = 0.04, *p* = 0.07). Moreover, neither was there any significant association between SkewnessH value and patient age (*r*^2^ = 0.003, *p* = 0.21), thus suggesting that degenerative osteoarthritis was not a confounding factor.

**Fig. 2:**
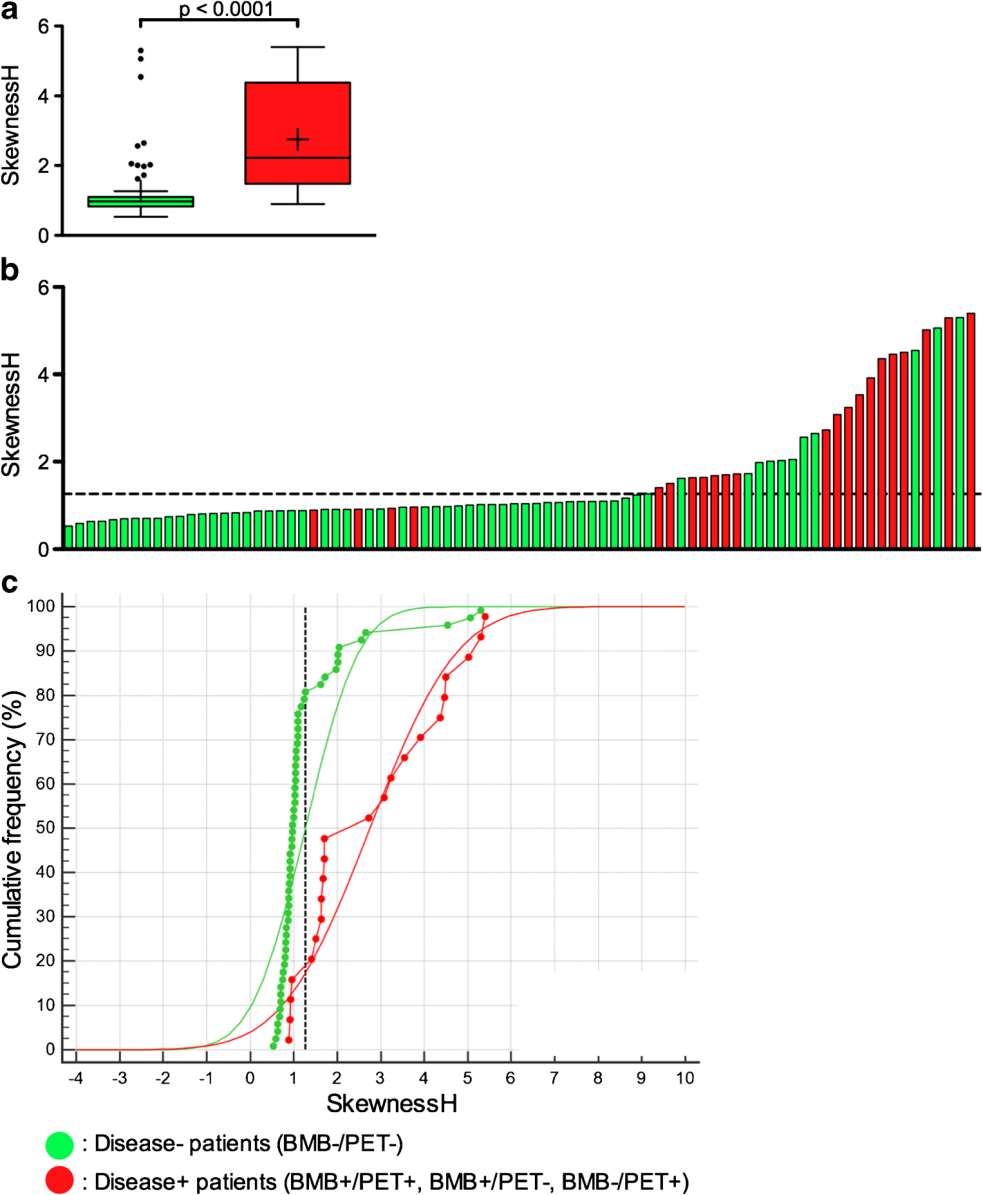
SkewnessH values of disease + and disease- patients. Data are shown as (a) Tukey boxplots (lines display the median, 25th and 75th percentiles; the cross represents the mean value), (b) histogram and (c) cumulative distribution functions

There was a significant difference between mean SkewnessH values extracted from disease-free patients’ images and those extracted from disease + patients’ images, with higher values in disease + patients: 2.75 ± 1.575 versus 1.26 ± 0.968, *p* < 0.0001 (Fig. 2). With a SkewnessH cut-off value set to 1.26, the sensitivity, specificity, positive predictive value, negative predictive value, positive likelihood ratio and negative likelihood ratio were 81.8%, 81.7%, 62.1%, 92.5%, 4.46 and 0.22, respectively. Fifty-three (64.6%) patients had a SkewnessH value ≤1.26 and 29 (35.4%) patients had a SkewnessH value >1.26 (Table 1). There were four SkewnessH false negative (FN) results (two BMB−/PET+ and two BMB+/PET+ patients) corresponding to two patients with unifocal abnormality on PET images and two patients with bone involvement outside the VOIs. There were also 11 false positive findings among the 60 BMB−/PET- patients. Notably, the unique BMB+/PET- patient with concordant bone involvement on BMB was efficiently diagnosed with BMI when using SkewnessH: she was a normal-weighted 58-year-old woman with a Bulky disease, an IPI score of 3 and SkewnessH value equal to 1.40667.

**Fig. 3:**
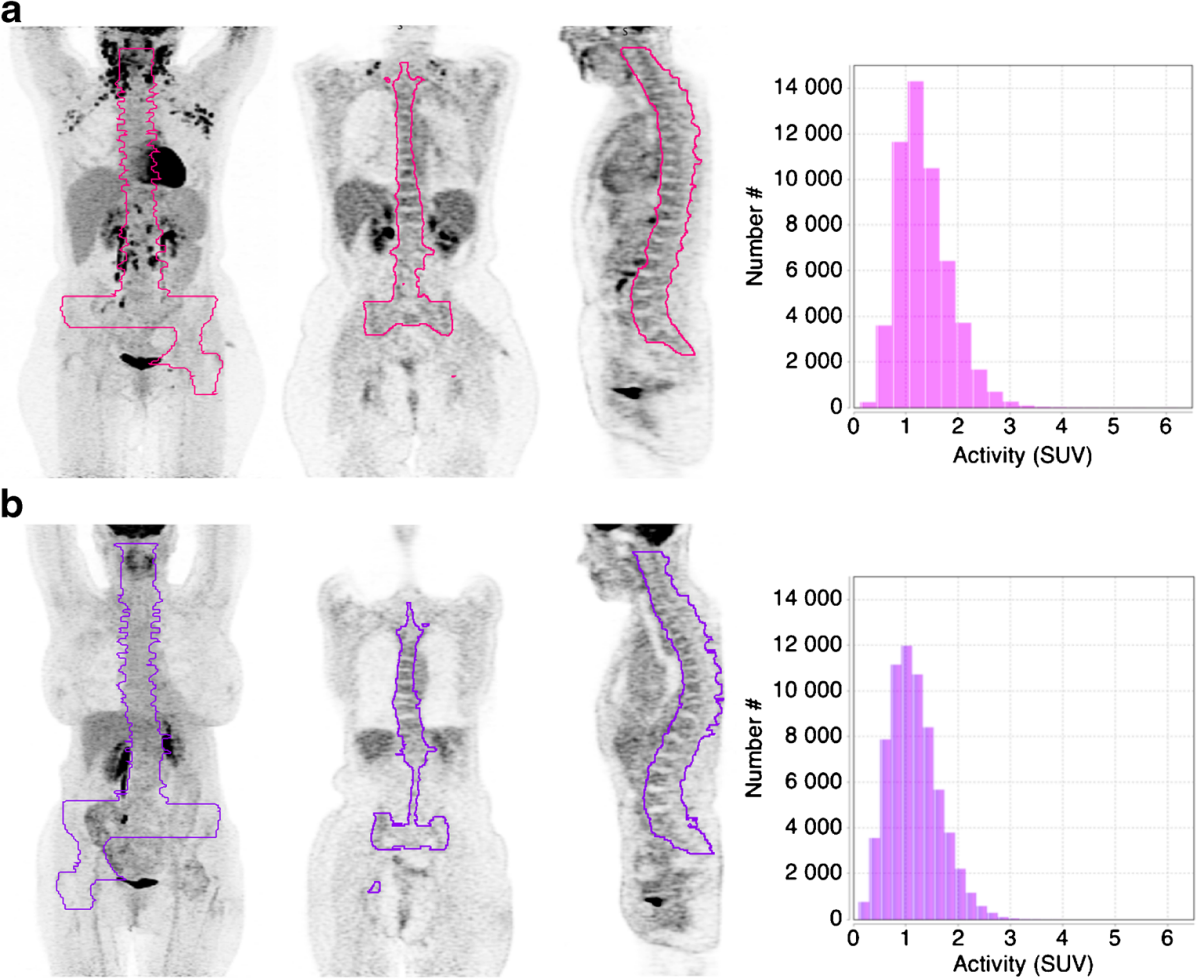
Representative images and VOI histograms of two BMB−/PET- patients. The patient displayed on panel (a) was considered positive for BMI according to PET textural feature assessment with a SkewnessH value equal to 5.30, whereas the patient displayed on panel (b) was considered negative with a Skewness value equal to 0.63. For each patient, from left to right, the maximum intensity projection (MIP) image, a coronal slice, a sagittal slice centred on the spine and the VOI histogram are displayed

Representative PET/CT images and corresponding VOIs of two BMB−/PET- patients with SkewnessH values ≤1.26 and >1.26 are shown in Fig. 3.

### Prognostic value of bone textural features at baseline staging

**Fig. 4:**
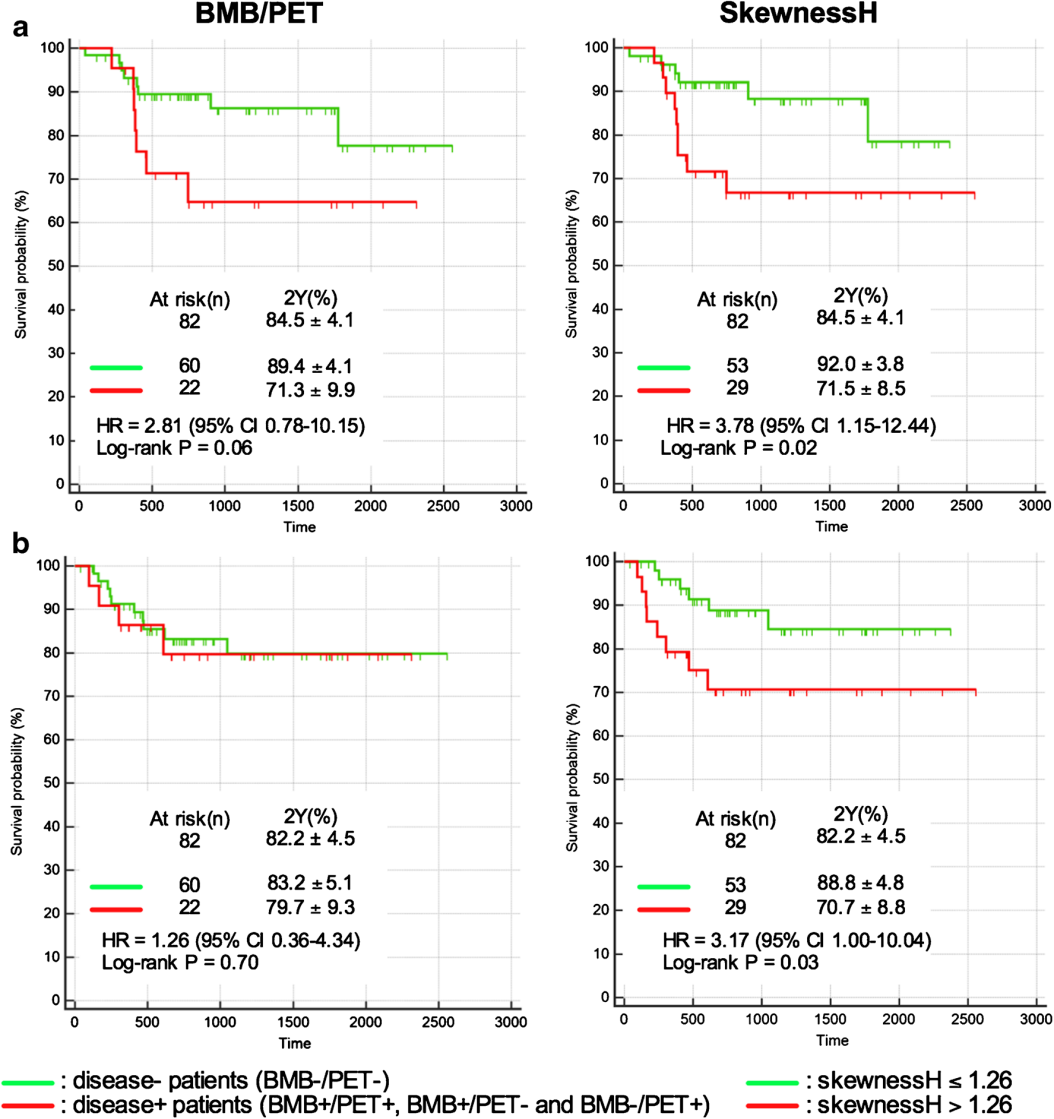
Prognostic value of BMB and visual PET assessment versus SkewnessH PET assessment. Panels (a) display overall survival (OS) curves and panels (b) progression-free survival (PFS) curves

**Fig. 5:**
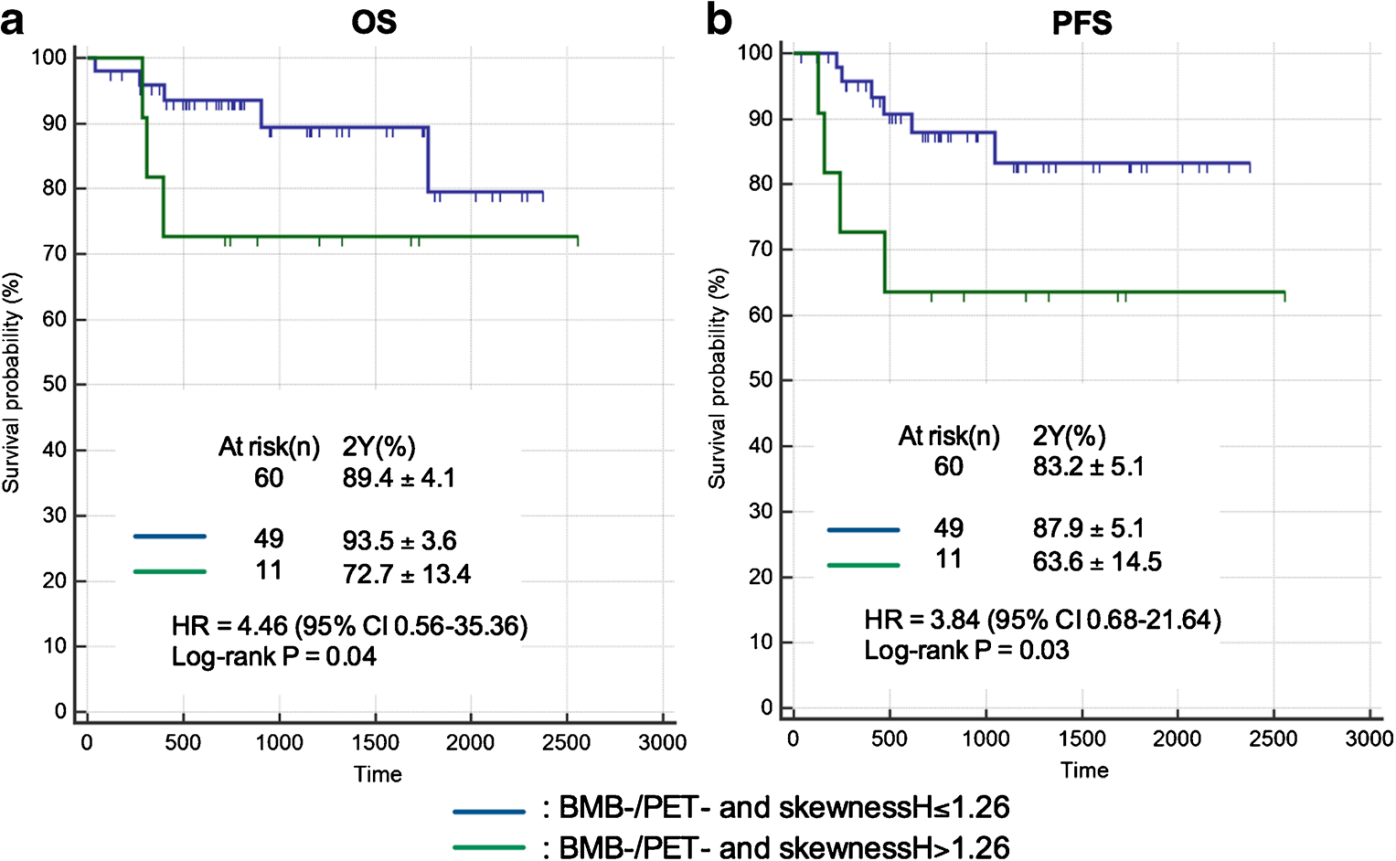
Prognostic value of SkewnessH PET assessment in disease- (BMB−/PET-) patients. Panel (a) displays overall survival curves and panel (b) progression-free survival curves

With a median follow-up of 25.7 months (range: 1.4 – 83.9 months), 13 patients (15.9%) experienced progression or relapse of their DLBCL, and 12 patients (14.6%) died from the lymphoma disease (lymphoma itself or treatment side-effects). For the whole population, the estimated PFS at 2 years was 82.2 ± 4.5%, and the estimated OS at 2 years was 84.5 ± 4.1%. In univariate analysis, using BMB results and PET bone marrow visual assessment, there was no significant difference between the PFS or OS of disease- (BMB−/PET-) patients and disease + (BMB−/PET+, BMB+/PET- and BMB+/PET+) patients (Fig. 4). Using the quantitative bone marrow assessment based on SkewnessH values, there was a difference between PFS and OS of SkewnessH negative patients (≤1.26) and SkewnessH positive patients (>1.26). The estimated PFS at 2 years was 88.8 ± 4.8% and 70.7 ± 8.8% for SkewnessH negative patients (≤1.26) and SkewnessH positive patients (>1.26), respectively (*p* = 0.03). The estimated OS at 2 years was 92.0 ± 3.8% and 71.5 ± 8.5% for SkewnessH negative patients (≤1.26) and SkewnessH positive patients (>1.26), respectively (*p* = 0.02) (Fig. 4). Notably, among 60 BMB−/PET- patients, there was a significant difference between the 2-year PFS of the 11 patients who had a SkewnessH value >1.26 and that of the 49 patients who had a SkewnessH value ≤1.26: 63.6% ± 14.5 and 87.9% ± 5.1, respectively (*p* = 0.03). For 2-year OS, similar result was observed (*p* = 0.04) (Fig. 5). In multivariate analyses integrating the international prognostic index (IPI), Bulky status, haemoglobin, and SkewnessH, the only independent predictor of OS was the IPI (Table 5), and the only independent predictor of PFS was SkewnessH (Table 6).

**Table 5:** Cox regression analysis for OS

	Univariate analysis			Multivariate analysis*		
Characteristics	HR	95% CI	P	HR	95%CI	P
SkewnessH >1.26	3.78	1.15-12.44	0.019	ns		
Bulky mass	2.12	0.67-6.74	0.188	–	–	–
IPI ≥ 2	5.31	1.62-17.40	0.016	5.34	1.16-24.55	0.032
Age > 60y	2.22	0.71-6.98	0.219	–	–	–
Stage III/IV	2.59	0.78-8.54	0.202	–	–	–
LDH+	9.22	2.24-38.03	0.0001	–	–	–
Hb < 12 g/dL	4.23	1.26-14.21	0.010	ns		

*Backward method, ns: not significant (*p* > 0.1)

**Table 6:** Cox regression analysis for PFS

	Univariate analysis			Multivariate analysis		
Characteristics	HR	95% CI	P	HR	95%CI	P
SkewnessH >1.26	3.17	1.00-10.04	0.032	–	–	–
Bulky mass	2.49	0.81-7.60	0.096	–	–	–
IPI ≥ 2	1.88	0.63-5.59	0.259	–	–	–
Age > 60y	2.43	0.81-7.33	0.161	–	–	–
Stage III/IV	1.65	0.52-5.25	0.439	–	–	–
LDH+	3.04	0.72-12.82	0.051	–	–	–
Hb < 12 g/dL	2.61	0.80-8.46	0.073	–	–	–

### Inter-observer VOI agreement

**Fig. 6:**
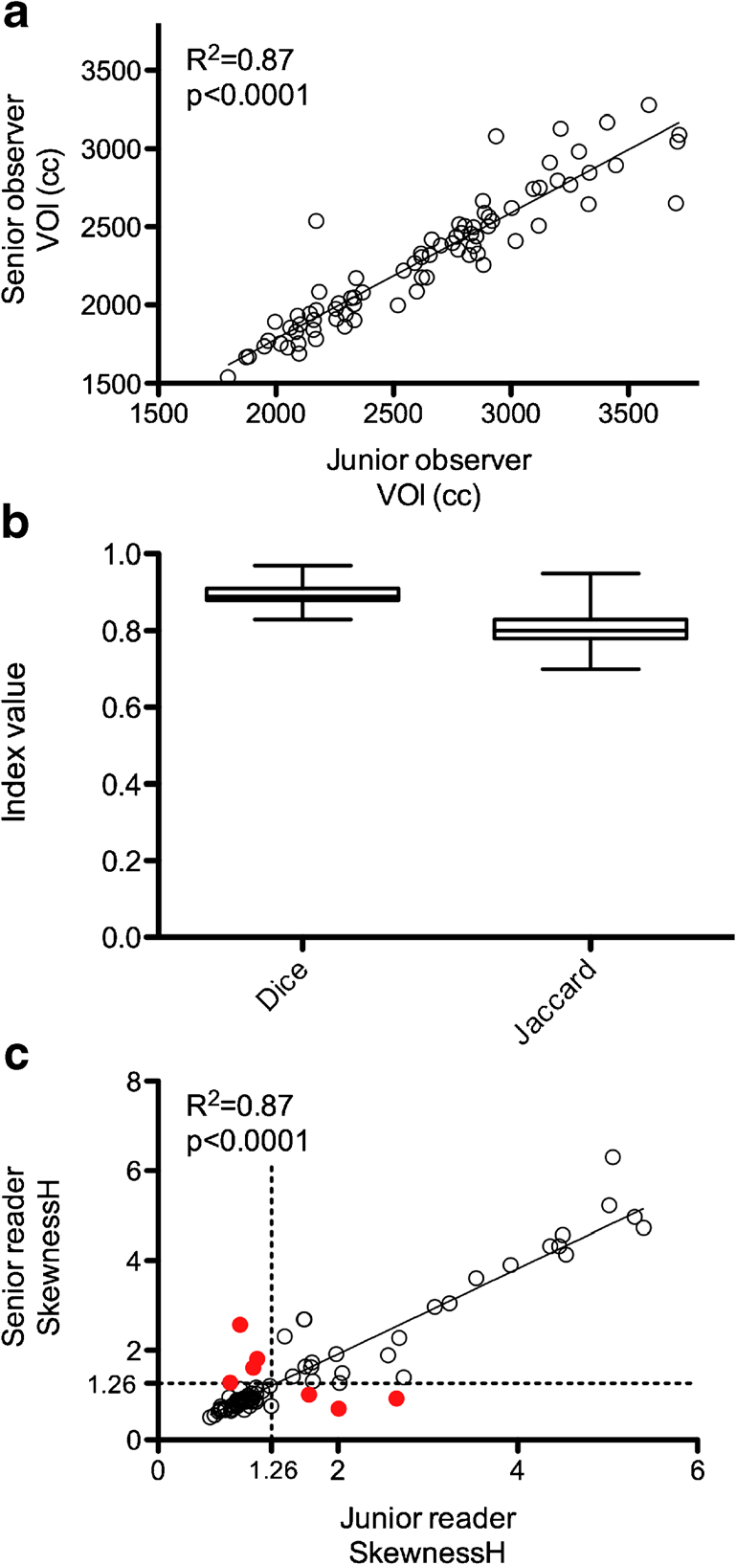
VOIs inter-rater agreement. Panel (a) displays the linear regression analysis for the VOIs, panel (b) displays spatial concordance indexes values as Tukey boxplots (lines display the median, 25th and 75th percentiles) and panel (c) displays the linear regression analysis for SkewnessH values

Linear regression showed a good agreement of VOI volumes (cc) between observers with a *r*^2^ value equal to 0.87 (*p* < 0.0001) (Fig. 6a). Moreover, spatial concordance of VOIs was almost perfect with mean Dice and Jaccard indexes equal to 0.89 ± 0.02 and 0.81 ± 0.04, respectively (Fig. 6b). Concerning SkewnessH values, there was also a good agreement between observers with a *r*^2^ value equal to 0.87 (*p* < 0.0001) (Fig. 6c). Inter-rater agreement for the diagnosis of BMI using SkewnessH was very good with κ value of 0.81 (95%CI = 0.68-0.95).

## Discussion

In the present study, conducted to determine the diagnostic and prognostic value of FDG skeletal TFs in DLBCL, the parameter displaying the better AUC and Youden index in diagnostic ROC analyses appeared to be SkewnessH. It is noteworthy that this metric is a first-order TF parameter, meaning that it is one of the simplest ones based on the VOI histogram [23]. Further ROC analyses with OS and PFS data as the reference standard were done and it was found also that first-order metrics performed better than second-order and third-order ones. However, this method had the disadvantage of giving different cut-off values for OS and PFS survival analyses. Using BMB and PET as reference, SkewnessH ROC analysis give a unique cut-off value set to 1.26. Using this cut-off, the sensitivity and specificity in detecting BMI were equal to 81.8% and 81.7%, respectively. Four false negative (FN) findings were observed among the 22 disease + patients (BMB+/PET+, BMB+/PET- and BMB−/PET+ patients). Two of them were related to BMB- patients harbouring small and unifocal PET abnormalities. These patients demonstrated good PFS and OS with no relapse or death at 2 years. Therefore, one could wonder about the clinical relevance of such findings. The two remaining FN findings were due to consequent bone involvement but outside the VOI. Due to technical considerations, more exhaustive VOIs could not yet be applied. Nevertheless, efforts could certainly be made on software development to figure out this issue. However, in clinical practice, the problem is currently mainly focused on negative PET scans for which BMI cannot be definitely excluded. Indeed, it was previously estimated in a recent meta-analysis that the proportion of patients in whom BMB is positive while FDG PET is negative for BMI reached 3.1% of cases. One could assume that this proportion of visually PET negative patients having actual BMI is certainly under-estimated because of the randomly selected and restricted BMB exploration, limited to a small zone of the pelvic bone. Previous studies had shown that discrepancy between biopsy sites may occur in as many as 10% to 60% of non-Hodgkin lymphomas [24–27]. In our study, 18.3% of BMB−/PET- patients (11out of 60 patients) were considered positive for BMI using SkewnessH (false positive findings). Moreover, these patients demonstrated worse PFS and OS than BMB−/PET- and SkewnessH negative patients at 2 years. This result has to be confirmed in larger studies, but it suggests that low-volume involvement of the bone marrow can be overlooked by both BMB and visual PET analysis and that bone heterogeneity assessment could help its diagnosis. Additionally, quantitative PET assessment of BMI on baseline FDG-PET/CT using SkewnessH demonstrated better discriminative power over visual PET assessment for the prognosis stratification of patients in our overall population and may be of some help in PET-negative patients for the diagnosis of low-volume BMI. In addition, SkewnessH appeared to be the only independent predictor of PFS in multivariate analysis.

A limitation of this study is that the drawing of the semi-automatic skeleton VOI is time consuming and that TF analysis has to be performed on third party software that is currently not approved for clinical use. Additionally, most of the TFs have been shown to be sensitive to reconstruction parameters, and the thresholds determined in the present study could not be used for patients scanned on other PET systems. With regard to this issue, our group has shown that some TFs such as entropy are less sensitive to reconstruction variability between PET centres [28], but SkewnessH was not tested in that previous study. However, Shiri et al. [29] and Galavis et al. [30] found consistent findings on the sensitivity of SkewnessH to reconstruction settings. To overcome this problem, we previously demonstrated that harmonised PET data could be considered [28, 31]. Harmonised images were not used in the present study, as our PET centre has been EARL accredited since 2015, and we included patients scanned from 2008 to 2015 to ensure a sufficient follow-up period for the purpose of survival analysis. Of note, PSF reconstruction with no post-filtering step has been shown to have potential for more discriminative power in stratifying or ranking patients; therefore, future studies aiming at confirming our results should ideally be performed on images optimised for diagnosis (for instance with PSF or PSF + TOF and post filtering with a low kernel or no post filtering) as well as on images meeting harmonising standards. Perspectives other than testing FDG skeletal TFs on harmonised PET data in larger and multicentric series would be to investigate the diagnostic value of TFs in other lymphoma subtypes for which BMB is performed on a regular basis, such as Follicular lymphomas (FL) and Hodgkin lymphomas (HL). Depending on the additional diagnostic value of FDG skeletal TFs over visual assessment in these lymphoma subtypes, they might be of some help in obviating BMB in certain cases or for guiding the site of biopsy.

## Conclusion

The better discriminative power of skeletal heterogeneity for risk stratification, compared to BMB and PET visual analysis in the overall population and more specifically in BMB−/PET- patients, suggests that it can be useful to identify BMI overlooked by PET visual analysis and BMB. The diagnostic value of FDG skeletal TFs should be confirmed with harmonised PET data in larger and multicentric series and in other lymphoma subtypes for which BMB is performed on a regular basis to determine whether TFs might be of some help in obviating BMB in certain cases or guiding the site of biopsy.
